# Development and validation of knowledge, attitude and practice questionnaire for prevention of respiratory tract infections among Malaysian Hajj pilgrims

**DOI:** 10.1186/s12889-020-8269-9

**Published:** 2020-03-02

**Authors:** Mohammed Dauda Goni, Nyi Nyi Naing, Habsah Hasan, Nadiah Wan-Arfah, Zakuan Zainy Deris, Wan Nor Arifin, Tengku Mohammad Ariff Raja Hussin, Abdulwali Sabo Abdulrahman, Aisha Abubakar Baaba, Muhammad Rafie Arshad

**Affiliations:** 1 Department of Medical Microbiology and Parasitology, School of Medical Sciences, Universiti Sains Malaysia, 16150 Kubang Kerian, Kelantan Malaysia; 2 Faculty of Medicine, Universiti Sultan Zainal Abidin, Medical Campus, 20400 Kuala Terengganu, Malaysia; 3 Faculty of Health Sciences, Universiti Sultan Zainal Abidin, Gong Badak Campus, 21300 Kuala Nerus, Terengganu Malaysia; 4 Unit of Biostatistics and Research Methodology, School of Medical Sciences, Universiti Sains Malaysia Health Campus, 16150 Kubang Kerian, Kelantan Malaysia; 5 Centre for Language Studies and Generic Development, Universiti Malaysia Kelantan, Locked Bag 01, 16300 Bachok, Kelantan Malaysia; 6 School of Computer Science, Universiti Sains Malaysia, 11800 Penang, Malaysia

**Keywords:** Item response theory, Exploratory factor analysis, Reliability, Knowledge attitude and practice, Respiratory tract infections and Hajj

## Abstract

**Background:**

Hajj pilgrimage faces numerous challenges including a high prevalence of respiratory tract infection as well as its prevention strategies. The aim of this study was to develop and validate a questionnaire to evaluate knowledge, attitude and practice (KAP) towards respiratory tract infections (RTIs) prevention among Malaysian Hajj pilgrims.

**Methods:**

This study was conducted among Malaysian Umrah pilgrims in Malaysia from Kuala Lumpur and Kelantan. The questionnaire then underwent a series of validation process that included content, face validity and exploratory part. Item response theory (IRT) analysis was utilized for the validation of the knowledge domain. The attitude and practice were validated using the exploratory factor analysis (EFA).

**Results:**

The validation process resulted in a questionnaire that comprised of four main sections: demography, knowledge, attitude, and practice. Following IRT analysis of the knowledge domain, all items analyzed were within the acceptable range of difficulty and discrimination. The Kaiser-Meyer-Olkin measure of sampling adequacy (KMO) was 0.72 and 0.84 for attitude and practice domain respectively and Bartlett’s test of Sphericity for both domains were highly significant (*P* < 0.001). The factor analysis resulted in two factors with total of 12 items in attitude domain, and 2 factors with total of 13 items in the practice domain with satisfactory factor loading (> 0.3). The Cronbach’s alpha for reliability of the knowledge, attitude and practice domains all showed acceptable values of > 0.6 (0.92, 0.77 and 0.85).

**Conclusion:**

The findings of this validation and reliability study showed that the developed questionnaire had a satisfactory psychometric property for measuring KAP of Malaysian Hajj pilgrims.

## Background

Annually, over 2 million Muslim pilgrims across the globe including thousands from Malaysia set out on the holy pilgrimage of Hajj in the Kingdom of Saudi Arabia [1]. This event is characterized by high density of crowding and posed a potential risk for confined outbreaks as well as the spread of infectious agents to different parts of the world upon pilgrims return to their various countries. Consequently, respiratory tract infections are the leading cause of hospital admission during the pilgrimage [2, 3].

The usual basic procedure in KAP evaluation is the questionnaire [4]. For the past four decades, KAP research has been the main educational intervention strategy for respiratory diseases control across the globe [5]. Various researchers had reported that an individual’s level of KAP is linked to competent control and prevention of illness, response to medical treatment, and advancement of one’s individual health [6–9]. KAP level at lower standard had been one of the foremost pointers of poor health, unproductive health care practice, the drop of the disease screening rate, and unrealistic preventive behavior toward various infections in various settings [10, 11]. Similarly, reliability is similar with precision and shows the extent to which the measurement tool is reproducible by determining its internal consistency [12].

The World Health Organization (WHO), the US Centre for Disease Control and prevention (CDC) and Saudi’s health authority have recommended preventive behaviors and practices for mitigating the risk, impact and spread of respiratory tract infections among pilgrims during Hajj pilgrimage which include practicing hand hygiene, proper use of a face mask, cough etiquette, social distancing, and contact avoidance [13]. However, the prevalence of RTI during Hajj has continued to rise over the recent years among Malaysian and other pilgrims across the world [14, 15]. Currently, there is a paucity of standardized and validated measurement tool on its KAP towards prevention of RTIs during Hajj.

Therefore, this research was conducted to develop and validate an instrument meant to evaluate the knowledge, attitude and practice in terms of face validity, content validity, exploratory factor analysis and reliability towards prevention of respiratory tract infection among Malaysian Hajj pilgrims in order to understand and improve prevention strategies using health educational intervention.

### Methodology

#### Questionnaire development

The questionnaire development and factor analysis took place in two stages, the first stage consisted of the questionnaire development stage, and the second stage comprised of psychometric evaluation. The psychometric evaluation comprised Exploratory Factor Analysis (EFA) and Item Response Theory (IRT) analysis.

#### First stage: items and domains development

For the first stage, a meticulous study of the literature was conducted to discover available resources on KAP, as well as to identify relevant items and scales in existing questionnaires on prevention of respiratory tract infection during Hajj pilgrimage. In-depth interviews were conducted on Hajj pilgrims attending weekly Umrah and Hajj orientation courses to qualitatively explore their baseline knowledge of RTIs, its prevention and control, risk factors, its mode of transmission and signs and symptoms as well as their attitude towards the risk of contracting the infection and prevention practices such as the use of face mask, vaccination, hand hygiene, cough etiquette and social distancing/crowd avoidance. An interview guide was used during the in-depth interview, and it consists of 70 items that emerged from the literature review covering the three domains. The interviews were transcribed and analyzed using content analysis. The first draft of the questionnaire was judged by a panel of experts that included a Medical Doctor, statistician, microbiologist, nurse and educationist to validate its contents with the intended constructs and theories.

The conclusions from the interviews on the extent of knowledge among the respondents were then used to generate suitable domains for the questionnaire.

The knowledge section was developed based on the etiology, transmission, risk, complications and symptoms of RTI. However, the attitude section of the questionnaire was developed based on the theory of Health Belief Model (HBM) [16], while the practice was based on the preventive strategies for respiratory tract infections recommended by the World Health Organization and the Centre for Disease Control and Prevention. Based on this, individuals who have accurate knowledge of respiratory tract infections and perceived susceptibility to and results of the infections and are informed of the interests of taking preventive actions are more likely to make significant lifestyle choices to prevent the onset of infection during Hajj pilgrimage.

Content validity of the KAP questionnaire was conducted with panel of experts which consisted of an epidemiologist, a microbiologist, a health educationist and a medical statistician. The panel select the best items for clarity of the questions, accuracy of the knowledge, attitude and practice domains and interpretability. This panel also helped in identifying and judging the content validity (relevance, coverage and representativeness) of the items initially selected for inclusion in the questionnaire.

The original draft of the questionnaire was developed in English and then translated to the Malay language which is the official national language for easy administration to local participants. Therefore, the questionnaire was translated based the standard translation guidelines. It underwent two forward translations by a language communication expert and an epidemiologist into Malay. Subsequently, the two sets of translated Malay questionnaire were subjected to a backward translation into English by another language communication expert as well as an epidemiologist. The aim of this process was to recognize and harmonize the translated items from the original English version and to produce accurate Malay version of the questionnaire [17].

Face validity was conducted on 10 Umrah pilgrims to evaluate the understanding of a layman towards comprehending the questionnaire and determine how meaningful the items were to target participants [18]. The participants were requested to describe and evaluate every questionnaire item following the open-ended discussion. Their various responses and understanding regarding the questions, how it was presented (layout and setting) and lack of vagueness were assessed. A revised and finalized version of the questionnaire was produced from the findings of the face validation for use throughout the rest of the research.

The questionnaire is self-administered and consisted of open-ended and closed-ended questions and was well received by participants. There was a total of four domains in the final questionnaire consisting of 80 questions, the domains were (1) demography of participants; (2) knowledge of RTIs; (3) attitude towards RTIs; (4) prevention practices towards RTIs. Socio demographic characteristics studied included age, gender, race and marital status, occupational status, level of education, how many times have you performed Hajj or Umrah in the last 5 years, vaccinations history, and presence of comorbidities and presence of RTI prior to departure to Hajj. The main domains, theories covered as well as the choices of response in the questionnaire are shown in Table 1.

**Table 1:** KAP questionnaire on respiratory tract infections prevention during Hajj

Domains	No. of Items	Measurements	Response choices
Demography	14	Socio-demographic, occupation, level of education, history of previous hajj or Umrah in the last 5 years, comorbidities, history of vaccination	Open-ended, closed-ended, multiple-choice
Knowledge	29	Etiology, transmission, risk groups, signs and symptoms, complications, the use of PPE and prevention practices	Yes/No/I don’t know; 1 = correct answer, 0 = wrong/I don’t know
Attitude	12 (5 reverse scored items)	Prevention attitudes regarding RTI based on health belief model	1 = Strongly disagree, 2 = Disagree, 3 = Not sure, 4 = Agree, 5 = Strongly agree
Practice	14	Preventive and risk-reduction infection practices, including healthy life style, the use of facemask, vaccination, hand hygiene and cough etiquette	0 = Always, 1 = Occasional, 2 = Never, 3 = Not applicable

#### Second stage: validation

The data collection for this study was conducted from January 2018 to May 2018. In total, 350 participants were recruited from Kuala Lumpur and Kelantan through a multistage sampling method during the weekly Umrah orientation session organized by a private Hajj and Umrah tour company and then retrieved back at the end of the day’s session to explore the psychometric property of the measurement tool. First, a briefing was done to the participants about the study and informed consent was then obtained from the respondents who agreed to be involved in the study. The KAP questionnaire forms were given to each participant for self-administration. Data were analyse using R software version 3.5.0, in the R Studio environment. The level of significance was set at 0.05.

#### Item response theory

The sample size required for 2-PL IRT is unspecified, although some studies suggest a range from 100 to 500 samples [19, 20]. For this study, the sample size was 350 participants after adding expected 30% drop-out rate. The unidimensionality of the knowledge domain with responses in dichotomous output as either correct answer or wrong answer, two-parameter logistic item response theory (2-PL IRT) analysis, using the *ltm* package version 1.0.0 was employed to analyse the knowledge domain. An acceptable range of difficulty (− 3 to + 3) and discrimination (0.25 to infinity) will serve as the cut off value for the psychometric properties’ evaluation of the domain. Item fit was evaluated by the chi-square goodness-of-fit per item, and unidimensionality was analyzed by modified parallel analysis.

#### Exploratory factor analysis (EFA)

The sample size required for EFA is 2 to 5 participants per item based on a recommendation by Kline [21]. As the number of the items was intended to be 62, a sample size estimated was 260 participants. The EFA was done to determine the construct validity of the attitude and practice domains of the questionnaire due to their ordinal responses. Kaiser-Meyer-Olkin measure of the sampling adequacy (KMO) and Bartlett’s test of the sphericity was done for sampling adequacy. The sample was considered adequate if the KMO value was more than 0.5 and Bartlett’s test was significant (*P* < 0.001). The Principal axis factoring method for the component extraction was used. Oblimin rotation with Kaiser normalization was applied in order to optimize the loading factor of each item on the extracted components. Dimensionality of the items in each domain was considered as continuous output and to facilitate its evaluation [22]. Components with Eigen values of over one was retained as components using parallel analysis and scree plot. Items with a loading factor of more than plus or minus 0.3 were considered as an acceptable loading factor [23].

#### Reliability

In this study the internal consistency (IC) of the items was measured by using Cronbach’s alpha coefficient. The items of the Questionnaire were considered to represent a measure of good internal consistency if the total of Cronbach’s alpha value was more than 0.7 [24].

## Results

### Questionnaire development and content and face validity

The extensive literature review on RTIs identified various useful concepts and ideas in generating important items and domains for the relevant sections of the questionnaire. Following an extensive review of the questionnaire by the panel, they collectively concurred that the incorporated domains and items were appropriate and consistent with the intended domains. The in-depth interview was conducted to aid the development of appropriate domains and items for incorporation in the questionnaire. This process assisted in distinguishing the items and local languages relating to respiratory tract infection (RTI) that were meaningful to the Hajj and Umrah pilgrims. Subsequently, the questionnaire was pretested by another session of in-depth interview on Umrah pilgrims during weekly orientation course to determine its face validity. Based on their opinions, most of wordings and terminologies were clear and easy to understand, however, the very few confusing ones were changed for easy understanding. Overall, they had no difficulty in understanding the items. The final draft of the questionnaire at this stage contained 4 domains and 68 items (14 items on demography, 29 items on knowledge, 12 items on attitude and 13 items on practice).

### Descriptive statistics of the participants

The mean age of the respondents was 43.85 (SD = 15.80) years with more than half (62.6%) being females as shown in Table 2. However, majority of the respondents were of Malay ethnicity (98.7%) with more than half of them married (67.4%).

**Table 2:** Socio-demographic characteristics of Umrah pilgrims (*n* = 350)

Variables	Mean (SD)	Frequency	Percentage (%)
Age	43.85 (15.80)		
Gender			
Male		110	37.4
Female		184	62.6
Ethnicity			
Malay		289	98.7
Chinese		0	0
Indian		1	0.3
Others		3	1.0
Marital status			
Single		85	28.9
Married		198	67.4
Divorced/widowed		11	3.7
Occupation			
Student		20	6.8
Civil servant		76	26.0
Private sector		92	31.5
Pensioner		45	15.6
Housewife		32	10.9
Self-employed		27	9.2
Highest level of education			
PhD		4	1.4
Master’s degree		20	6.8
Bachelor’s degree		58	19.7
Diploma		93	31.6
Secondary school		107	36.4
Primary school		12	4.1
History of vaccination			
Meningococcal vaccine		111	37.8
Influenza (flu) vaccine		52	17.7
Pneumococcal vaccine		25	8.5
Presence of Co-morbidities			
Chronic lung disease		4	1.4
Neuromuscular disease		10	3.4
Allergic rhinitis		5	1.7
Diabetes		21	7.1
Hypertension		46	15.6
Heart disease		8	2.7
Chronic kidney disease		2	0.7
Immune deficiency disorders		1	0.3

### Item response theory

The knowledge section analysed by IRT analysis showed the psychometric properties of the domain as shown in Table 3. The sub-domains are Aetiology (K1i, K1ii, K1iii), transmission (K2i, K2ii, K2iii, K2iv, K2v, K3), risk factors (K4i, K4ii, K4iii, K4iv, K4v and K4vi), complications (K5i, K5ii, K5iii, K5iv and K5v), prevention practices (K6i, K6ii, K6iii, K6iv and K6v) and Use of personal protective equipment (K7i, K7ii, K7iii, K8 and K9). The difficulty parameter indicated that all the items were within the acceptable range of − 3 to + 3. With regards to the range of values for items for discrimination, items K1ii, K2i, K2iii, K2v, K3, K4i, K4v, K4vi, K6i, K6ii, K6iv, K8 and K9 were within the acceptable range of 0.8 to 2.5. as shown in Table 3 [25]. The item K19 was exempted from the IRT analysis because of its poor performance in the questionnaire. The remaining items were above the cut-off values. However, all the items that were above the range were retained based on expert advice due to the importance of their content in determining knowledge of the participants [20]. The item goodness-of-fit showed that all the items did not fit well with the exception of K1iv, K6iii, K6v and K7i (*P* < 0.05). Similarly, all questions in the knowledge domain were retained based on expert advice due to their importance and relevance to the study. The amount of information tapped by the items between − 3 and + 3 difficulty across the 6 sub-domains of the knowledge section ranged from 98.44 to 99.94%. The unidimensionality assumption of the items were supported by the modified parallel analysis in three knowledge sub-domains only namely: Aetiology, risk factors and prevention practices (*P* = 0.2376, 0.5248, 0.2574). Furthermore, the Cronbach’s alpha for all the sub-domains were demonstrating acceptable internal consistency reliability (0.80, 0.78, 0.79, 0.79, 0.84, 0.71, 0.71).

**Table 3:** Results of the IRT analysis in the knowledge section (*n* = 318)

Items	*b*	*a*	λ	χ^2^ (df = 8)	*P* values
K1 Flu-like illnesses are caused by:					
*Penyakit seperti selsema disebabkan oleh:*					
K1i Virus	−1.04	3.12	0.87	120.2	**< 0.001**
*Virus*					
K1ii Bacteria	−0.54	2.16	0.78	62.98	**< 0.001**
*Bakteria*					
K1iii Allergies	−0.41	3.50	0.9	100.55	**< 0.001**
*Alergi*					
K2 Flu-like illnesses are spread by:					
*Penyakit seperti selsema disebarkan oleh:*					
K2i Water	0.24	2.32	0.82	88.76	**< 0.001**
*Air*					
K2ii Sharing towels with an infected person	−0.32	3.95	0.94	52.78	**< 0.001**
*Berkongsi tuala dengan penghidap*					
K2iii Dust	−0.86	2.24	0.79	26.19	**0.001**
*Debu*					
K2iv Air	−1.10	3.39	0.88	24.34	**0.002**
*Udara*					
K2v Shaking the hands of an infected person with a cough and/or cold	−0.16	1.90	0.75	52.42	**< 0.0001**
*Berjabat tangan dengan pengidap yang batuk atau demam*					
K3 Flu-like illnesses are spread quickly	−1.17	1.41	0.64	101.32	**< 0.001**
*Penyakit seperti selsema tersebar dengan pantas*					
K4 The following persons are at an increased risk of flu-like illnesses:					
*Berikut merupakan mereka yang berisiko tinggi mengalami penyakit seperti selsema:*					
K4i Senior citizens aged 65 and older	−0.57	1.94	0.75	29.26	**< 0.001**
*Warga emas berusia 65 tahun ke atas*					
K4ii Smokers	−0.14	3.08	0.87	75.46	**< 0.001**
*Perokok*					
K4iii Asthmatics	−0.87	2.83	0.86	43.63	**< 0.001**
*Pesakit asma*					
K4iv Diabetics	0.40	4.32	0.93	21.16	**0.007**
*Pesakit diabetes*					
K4v People with arthritis	0.43	2.34	0.80	50.84	**< 0.001**
*Penghidap artritis*					
Kvi Those in crowded places/among a lot of people	−1.13	1.83	0.73	49.71	**< 0.001**
*Mereka yang berada di tempat yang sesak/dalam kalangan ramai orang*					
K5 What are the complications of flu-like illnesses?					
*Apakah komplikasi penyakit seperti selsema?*					
K5i Pneumonia	−0.27	2.76	0.90	91.12	**< 0.001**
*Paru-paru berair*					
K5ii Bronchitis	−0.14	2.93	0.91	126.49	**< 0.001**
*Bronkitis*					
K5iii Difficulty in breathing	0.64	6.26	0.88	22.80	**0.004**
*Kekejangan*					
K5iv Multi-organ failure	0.55	2.85	0.89	170.57	**< 0.001**
*Kegagalan pelbagai organ*					
K6 The following practices can help protect you from flu-like illnesses:					
*Amalan berikut dapat menghindari anda daripada penyakit seperti selsema:*					
K6i Ensuring a healthy diet	−1.05	2.29	0.50	38.14	**< 0.001**
*Memastikan pemakanan yang sihat*					
K6ii Receiving vaccinations	−0.80	2.26	0.85	66.51	**< 0.001**
*Menerima vaksinasi*					
K6iii Washing your hands with hand sanitizers	−0.86	6.29	0.78	15.08	0.058
*Membasuh tangan dengan sanitasi*					
K6iv Covering your nose with your hands	−0.67	1.75	0.92	71.99	**< 0.001**
*Menutup mulut dengan tangan*					
K6v Wearing a face mask	−1.22	5.07	0.71	10.75	0.216
*Memakai topeng muka*					
K7 The following are reasons for wearing a mask:					
*Berikut merupakan sebab memakai topeng muka:*					
K7i Being in crowded places	−1.03	6.11	0.97	11.82	0.160
*Berada di tempat yang sesak*					
K7ii Being near people who are coughing	−1.26	4.83	0.96	20.75	**0.008**
*Berada berdekatan orang yang batuk*					
K7iii When I am sick	−0.91	4.33	0.94	49.03	**< 0.001**
*Apabila saya sakit*					
K8 A cloth facial mask is as effective as a 2-ply surgical facial mask	1.02	1.29	0.60	60.85	**< 0.001**
*Topeng wajah kain sama berkesan dengan topeng wajah pembedahan dua lapisan*					
K9 If I am not sick, the used face mask can be stored in a bag for later use	0.72	1.56	0.67	182.10	**< 0.001**
*Jika saya tidak sakit, topeng muka terpakai boleh disimpan dalam beg untuk kegunaan seterusnya*					

*a* discrimination, *b* difficulty, *df* degree of freedom, *IRT* item response theory, *SE* standard error, χ^2^chi-square, λ standardized loadingItems with *P* values < 0.05 in the assessment of the item fit are highlighted in bold

### Exploratory factor analysis

For the attitude domain, the items were subjected to EFA to examine the Kaiser-Meyer-Olkin (KMO) test and Bartlett’s test of sphericity were to evaluate the factorability. The KMO measure of sampling adequacy was 0.72 and the significance of Bartlett’s test of sphericity was less than 0.001, meaning that EFA could be applied [26]. Parallel analysis for attitude sub-domain suggested a 4-factor model, however EFA was proceeded by fixing the number of factors to two based on the eigenvalue greater than one criterion. Those factors obtained were rotated using Oblimin method to make the factors more meaningful which propitiously resulted to two interpretable factors based on the relation among the items and the proposed domain. All the items had acceptable factor loading of > 0.3 in the attitude domain with the exception of items A1, A2, and A12B and therefore they were removed.

The validity of the 2-factor model was further confirmed by the communalities of each attributes as all the communalities were close to one. The 2 domains are barriers to compliance (items A3, A8, A9, A10 and A11), and self-motivation (A4, A5A, A5B, A6, A7, A12A and A13) as shown in Table 4.

**Table 4:** Results of the EFA of the attitude domain

Factors	Items	Factor loading	Reliability (Cronbach’s alpha)
Barriers to compliance	A3: Since the bird flu, SARS, MERS-COV and H1N1 crises are over, I no longer need to worry about contracting flu-like illnesses	0.527	0.81
	*Sejak krisis selesema burung, SARS, MERS-COV and H1N1 berakhir, saya sudah tidak perlu bimbang mendapat penyakit mirip influenza (demam selesema/influenza like illness)*		
	A8: I am generally opposed to wearing a face mask	0.741	
	*Secara amnya saya menentang penggunaan penutup hidung dan mulut*		
	A9: Flu vaccinations have unpleasant side effects	0.641	
	*Vaksinasi influenza mengakibatkan kesan sampingan yang tidak selesa*		
	A10: I am influence by negative news about flu vaccines	0.791	
	*Saya dipengaruhi oleh berita negatif mengenai vaksin selsema*		
	A11: It is too much trouble to get a flu vaccine	0.662	
	*Agak susah untuk mendapatkan vaksin influenza*		
Self-motivation	A4 If I have a flu-like illness, I may spread it to others	0.58	0.70
	*Jika saya menghidap penyakit mirip influenza (demam selesema/influenza like illness), saya mungkin menjangkiti orang lain*		
	A5: I feel that someone that have influenza-like illness should:		
	*Seseorang yang mengalami penyakit mirip influenza (demam selesema/influenza like illness) haruslah*:		
	A5A: cover his mouth and nose with his bare hand when coughing or sneezing	0.462	
	*Menutup mulut dan hidung dengan tangannya apabila batuk atau bersin*		
	A5B: cover his mouth and nose with a handkerchief when coughing or sneezing	0.414	
	*Menutup mulut dan hidung dengan sapu apabila batuk atau bersin*		
	A6: Influenza vaccines protects hajj pilgrims from influenza	0.568	
	*Vaksin influenza melindungi jemaah haji daripada demam selesema*		
	A7: Using a hand wash can prevent you from getting flu like illness	0.434	
	*Penggunaan sanitasi tangan dapat mencegah anda daripada menghidap penyakit mirip influenza (demam selesema/influenza like illness)*		
	A12A: I think coughs and the flu can be prevented by wearing a mask outside my house	0.588	
	*Saya rasa penyakit mirip influenza (demam selesema/influenza like illness) boleh dicegah dengan pemakaian sungkup muka di luar rumah*		
	A13: Wearing a well-fitting face mask is effective in preventing flu-like illnesses	0.414	
	*Memakai sungkup muka yang betul-betul sesuai pada wajah adalah berkesan untuk mencegah penyakit seperti selsema*		

For practice domain, the data matrix was factorable and assumptions to conduct EFA were met as indicated by a KMO value of 0.84 and Bartlett’s test of sphericity being significant (*P* = 0.005). The EFA was continued by fixing the number of factors to two as suggested by parallel analysis. All the items in the practice section had factor loading of more than 0.3 and were retained with the exception of item P8D which was removed due to cross loading. The two sub-domains factored are healthy life-style (items P1, P2 and P5) and prevention practices (P4, P6, P7, P8A, P8B, P8C, P9A, P9B, P9C and P10) as shown in Table 5.

**Table 5:** Results of the EFA of the practice domain

Factors	Items	Factor loading	Reliability (Cronbach’s alpha)
Health lifestyle	P1: I eat vegetables	0.917	0.83
	*Saya makan sayur*		
	P2: I eat fruits	0.878	
	*Saya makan buah-buahan*		
	P5: I use soap to wash my hands	0.556	
	*Saya menggunakan sabun untuk membasuh tangan saya*		
Prevention practices	P4: When wearing a mask, I test it to ensure it fits properly	0.433	0.83
	*Ketika memakai penutup hidung dan mulut (face mask), saya memastikan ianya benar-benar kemas*		
	P6: I use disinfectant or disposable wipes or hand gel to wash my hands	0.543	
	*Saya menggunakan penyahinfektan atau pengelap pakai buang atau gel tangan untuk membasuh tangan saya*		
	P7: I use a washable cloth handkerchief to clean my hands	0.315	
	*Saya menggunakan sapu tangan yang boleh dibasuh untuk membasuh tangan saya*		
	P8: I wash my hands after:		
	*Saya membasuh tangan saya:*		
	P8A: touching the personal items of someone who has a cough and/or cold	0.67	
	*Selepas menyentuh barang peribadi seseorang yang mengalami batuk atau selsema*		
	P8B: shaking hands with people who have a cough and/or cold	0.769	
	*Selepas berjabat tangan dengan seseorang yang mengalami batuk atau selsema*		
	P8C: touching door knobs	0.679	
	*Selepas menyentuh tombol pintu*		
	P9: I refrain from:		
	Saya menghindari daripada:		
	P9A: being close to those who cough or sneeze	0.571	
	*berada rapat dengan orang yang batuk atau selesema*		
	P9B: shaking the hands of those who have a cough and/or cold	0.671	
	*berjabat tangan dengan orang yang batuk atau selesema*		
	P9C: often touching my nose	0.474	
	*kerap menyentuh hidung saya*		
	P10: I received the flu vaccine	0.486	
	*Saya menerima vaksin selsema*		

### Reliability

In order to measure the reliability, the Cronbach’s alpha of the overall questionnaire was 0.92, 0.60, 0.86 for knowledge, attitude and practice sections respectively. For the attitude domain, the two factors (barriers to compliance and self-motivation) have acceptable internal consistency (0.81 and 0.70). Similarly, the practice domain also has an acceptable value for Cronbach’s alpha of 0.83 for both sub-domains (health life-style and prevention practices) as shown in Table 5.

## Discussion

To the best of our knowledge, this is the first study that describes the development of a validated questionnaire with satisfactory content and face validity and reliability examining the knowledge, attitude and practice of Malaysian Hajj pilgrims towards prevention of respiratory tract infections. The present study reported the stages of designing and developing a questionnaire for determining the knowledge, attitude and practice of Hajj pilgrims from Malaysia towards prevention of respiratory tract infections indicated satisfactory psychometric properties for the questionnaire.

Based on the content validity evaluation of the questionnaire, some items were deleted as they were shown to be problematic validity in terms of their relevance in measuring KAP of prevention of respiratory tract infections. Performing both exploratory factor analyses, the results indicated a good structure for this new instrument. The IRT on the knowledge section showed a good difficulty psychometric property of the domain. The ideal parameter range for discrimination value range from minus infinity to plus infinity; nonetheless, questions with negative figures of discrimination are recognized as problematical because they infer that participants with a high score are less expected to support more stringent response alternatives [27]. Our findings showed all discrimination parameters are positive and less problematic as seen in various studies [28].

For the attitude section, EFA indicated a two-factor structure of the questionnaire could jointly account for 72.3% of the total observed variance which was what was hypothesized. All factor loadings were above 0.3, revealing close relations between factors and items [29]. The reliability analysis of the attitude section indicated acceptable Cronbach’s alpha value demonstrating internal consistency. In the practice section, the analysis resulted in a good-fitting two-factor model as well, with good reliability and internal consistency (> 0.6). The two- factor model extracted in EFA for this domain explained 62.52% of the total variance, which was higher than the criterion of 50%.

The limitation of this study was that item response theory and exploratory factor analysis was used to assess the reliability and validity, however, it is recommended that a confirmatory factor analysis should be conducted in the future to validate the knowledge, attitude and practice questionnaire of respiratory tract infection prevention.

## Conclusion

In this study, a new validated questionnaire for determining KAP of respiratory tract infection prevention in Malay language was developed among samples Malaysian Hajj pilgrims. The final questionnaire consisted of 4 sections and 76 items (14 items on demography and general information, 29 items on knowledge domains, 12 items in attitude and 14 items in practice domain. The knowledge, attitude and practice sections showed acceptable psychometric properties and has good reliability result.

## Data Availability

The datasets used and/or analysed during the current study are available from the corresponding author on reasonable request.

## Abbreviations

EFA: Exploratory factor analysis
HBM: Health Belief Model
IRT: Item response theory
KAP: Knowledge, attitude, and practice
KMO: Kaiser-Meyer-Olkin
RTI: Respiratory tract infection
